# Supplementary material for: A computational study on the role of glutamate and NMDA receptors on cortical spreading depression using a multidomain electrodiffusion model
Source: PLoS Comput Biol. 2019 Dec 2;15(12):e1007455. doi: 10.1371/journal.pcbi.1007455 (PMC6907880; doi:10.1371/journal.pcbi.1007455)
Supplement: S1 Text — Here, details of the ion channel models as well as the parameters used in the simulations are listed. (PDF) [file pcbi.1007455.s001.pdf]

Supporting Information 1 for: A computational study on the role of glutamate and NMDA receptors on cortical spreading depression using a multidomain electrodiffusion model

Austin Tuttle Jorge Riera-Diaz Yoichiro Mori

S1: Details of Model

S1a: Neuronal Ion Channels

The different neuronal ion channel models are summarized in Table 1. NaK ATPase flux is expressed in terms of h_{NaK}, which is computed as follows:

$$h^{\mathrm{n}}_{\mathrm{NaK}} = \frac{I^{\mathrm{n}}_{\max}}{(1 + m_{\mathrm{K}}/c^{\mathrm{e}}_{\mathrm{K}})^2(1 + m_{\mathrm{Na}}/c^{\mathrm{n}}_{\mathrm{Na}})^3}.$$

Here, 1.5972×10^{-7}mmol/cm^2/s, $m_{\mathrm{K}} = 2$mmol/l and $m_{\mathrm{Na}} = 7.7$mmol/l.

S1b: Glial Ion Channels

Na and Cl leak fluxes have expressions that are identical to those of neurons. In glia, the Potassium leak channel is replaced by the Potassium inward rectifier (KIR), whose flux expression is given below.

$$\hat{g}^{\mathrm{g,IR}}_{K} = \sqrt{\frac{c^{\mathrm{e}}_{\mathrm{K}}}{3}}\frac{1 + \exp(18.5/42.5)}{1 + \exp((\phi_{\mathrm{ge}} - E^{\mathrm{g}}_{\mathrm{K}} + 18.5)/42.5)}\frac{1 + \exp((-118.6 - 85.2)/44.1)}{1 + \exp((-118.6 + \phi_{\mathrm{ge}})/44.1)}$$

$$J^{\mathrm{g,IR}}_{\mathrm{K}} = \left(\phi_{\mathrm{ge}} - E^{\mathrm{g}}_{\mathrm{K}}\right)$$

$$P^{\mathrm{g,IR}}_{\mathrm{K}} = 1.34736 \times 10^{-7} mSmol/cm^2C$$

$$j_{\mathrm{KIR}} = P^{\mathrm{g,IR}}_{\mathrm{K}}\hat{g}^{\mathrm{g,IR}}_{\mathrm{K}}J^{\mathrm{g,IR}}_{\mathrm{K}}$$

In the above, $E^{\mathrm{g}}_{\mathrm{K}}$ is the Nernst-Potential in the glial compartment for potassium and $\hat{g}$ is the percentage of channels open.

NaK ATPase is the same as in neurons, except that we set $I^{\mathrm{g}}_{\max} = 7.5890 \times 10^{-8}$ mmol/cm^2/s. The expression for the NaKCl cotransporter is given by:

$$J^{\mathrm{NaKCl}}_{\mathrm{g}} = P^{\mathrm{NaKCl}} \ln\left(\frac{c^{\mathrm{g}}_{\mathrm{Na}}c^{\mathrm{g}}_{\mathrm{K}}(c^{\mathrm{g}}_{\mathrm{Cl}})^2}{c^{\mathrm{e}}_{\mathrm{Na}}c^{\mathrm{e}}_{\mathrm{K}}(c^{\mathrm{e}}_{\mathrm{Cl}})^2}\right)$$

$$h^{\mathrm{g,NaKCl}}_{\mathrm{Na}} = J^{\mathrm{NaKCl}}_{\mathrm{g}}$$

$$h^{\mathrm{g,NaKCl}}_{\mathrm{K}} = J^{\mathrm{NaKCl}}_{\mathrm{g}}$$

$$h^{\mathrm{g,NaKCl}}_{\mathrm{Cl}} = 2J^{\mathrm{NaKCl}}_{\mathrm{g}}$$

where, $P^{\mathrm{NaKCl}} = 9.1806 \times 10^{-10}$ mmol/cm^2/s.

Currents	Type	Gates($m^p h^q$)	Voltage-Dependent Rate Constants
I_{NaT}	GHK	m^3h	$\alpha_m = 4\varphi(.25\phi + 12.975)$<br>$\beta_m = 1.4\hat{\varphi}(.2\phi + 4.978)$<br>$\alpha_h = .128\exp(-(0.056\phi + 2.94))$<br>$\beta_h = \frac{4}{1+\exp(-(.2\phi+6))}$
I_{NaP}	GHK	m^2h	$\alpha_m = \frac{1}{6(1+\exp(-(.143\phi+5.67)))}$<br>$\beta_m = \frac{\exp(-(.143\phi+5.67))}{6(1+\exp(.143\phi+5.67))}$<br>$\alpha_h = 5.12 \times 10^{-8}\exp(-(.056\phi + 2.94))$<br>$\beta_h = \frac{1.6\times10^{-6}}{1+\exp(-(.2\phi+8))}$
I_{KDR}	GHK	m^3	$\alpha_m = 0.08\varphi(0.2\phi + 6.98)$<br>$\beta_m = 0.25\exp(-(0.25\phi + 1.25))$
I_{KA}	GHK	m^2h	$\alpha_m = 0.2\varphi(0.1\phi + 5.69)$<br>$\beta_m = 0.175\hat{\varphi}(0.1\phi + 2.99)$<br>$\alpha_h = 0.016\exp(-(0.056\phi + 4.61))$<br>$\beta_h = \frac{0.5}{1+\exp(-(0.2\phi+11.98))}$
I_{NaL}	HH	None	
I_{KL}	HH	None	
I_{ClL}	HH	None	

Table 1: Neuronal ion channel models. In the above, $\varphi(u) = u/(1 - \exp(-u))$ and $\hat{\varphi}(u) = u/(\exp(u) - 1)$.

S1c: Other Parameters in Model

A table of constants used in the electrodiffusion model are given in Table 2 (Values come from [5, 4, 1]).

References

[1] H. Kager, W. Wadman, and G. Somjen, *Simulated seizures and spreading depression in a neuron model incorporating interstitial space and ion concentrations*, Journal of neurophysiology, 84 (2000), pp. 495–512.

[2] C. Nicholson, *Diffusion and related transport mechanisms in brain tissue*, Reports on progress in Physics, 64 (2001), p. 815.

[3] R. O'Connell and Y. Mori, *Effects of glia in a triphasic continuum model of cortical spreading depression*, Bulletin of mathematical biology, 78 (2016), pp. 1943–1967.

[4] Y. Wei, G. Ullah, and S. J. Schiff, *Unification of neuronal spikes, seizures, and spreading depression*, Journal of Neuroscience, 34 (2014), pp. 11733–11743.

Paramater	Value
General	
D_{Na}	1.33×10^{-5} cm^2/sec
D_{K}	1.96×10^{-5} cm^2/sec
D_{Cl}	2.03×10^{-5} cm^2/sec
Tortuosity(λ)	$1.2 - 2.4$[2], we use 1.6
C_{m}	0.75×10^{-3} mF/cm^2
$1/\gamma_k, k = \mathrm{e}, \mathrm{g}$	1.566×10^{-4}cm
$\eta_k, k = \mathrm{e}, \mathrm{g}$	5.4×10^{-5} cm/sec/(mmol/cm^3)
m_{K}	2×10^{-3} mmol/cm^3
m_{Na}	7.7×10^{-3} mmol/cm^3
R	8.314472×10^6nJ/K/mmol
T	310.15K
F	$9.64853399 \times 10^7 \mu$C/mmol
Neuron	
P_{NaP}	2×10^{-5} cm/s
P_{KDR}	1×10^{-3} cm/s
P_{KA}	1×10^{-4} cm/s
$P^{\mathrm{n}}_{\mathrm{NaL}}(*)$	6.2695×10^{-9}cm/s
P_{KL}	7×10^{-2} mS/cm^2
$P^{\mathrm{n}}_{\mathrm{ClL}}$	10×10^{-2} mS/cm^2
$I^{\mathrm{n}}_{\mathrm{max}}(*)$	1.5972×10^{-7}mmol/cm^2/s
Glia	
P_{KIR}	0.13 mS/cm^2
P_{NaKCl}	9.1806×10^{-10} mmol/cm^2/s
$P^{\mathrm{g}}_{\mathrm{NaL}}(*)$	2.1290×10^{-9}cm/s
$P^{\mathrm{g}}_{\mathrm{ClL}}$	5×10^{-2} mS/cm^2
$I^{\mathrm{g}}_{\mathrm{max}}(*)$	7.5890×10^{-8} mmol/cm^2/s

Table 2: Lis of constants in the model. The values with an asterisk are calculated so that the ionic concentrations and voltages at steady state take appropriate physiological values. For each compartment, the diffusion coefficient is calculated as follows. For the extracellular space, we set $D_i^{\mathrm{e}} = \alpha_{\mathrm{e}} D_i/\lambda^2$ where λ is the tortuosity as defined above. For Neurons, the diffusion coefficient is set to 0 (no gap junctional coupling). For Glia, we set $D_i^{\mathrm{g}} = d_{\mathrm{g}} D_i/\lambda^2$ where $d_{\mathrm{g}} = 3/40$, where this number is based on simulations in [3].

[5] W. Yao, H. Huang, and R. M. Miura, *A continuum neuronal model for the instigation and propagation of cortical spreading depression*, Bulletin of mathematical biology, 73 (2011), pp. 2773–2790.
