# Supplementary material for: A computational study on the role of glutamate and NMDA receptors on cortical spreading depression using a multidomain electrodiffusion model
Source: PLoS Comput Biol. 2019 Dec 2;15(12):e1007455. doi: 10.1371/journal.pcbi.1007455 (PMC6907880; doi:10.1371/journal.pcbi.1007455)
Supplement: S2 Text — A description of the calculation of velocity, duration and energy expenditure is given. (PDF) [file pcbi.1007455.s002.pdf]

# Supporting Information 2 for: A computational study on the role of glutamate and NMDA receptors on cortical spreading depression using a multidomain electrodiffusion model

Austin Tuttle Jorge Riera-Diaz Yoichiro Mori

## S2: Specifics of Calculation

### S2a: Velocity

Assume we have a function $F(x, y, t) : [0, L]^2 \times \mathbb{R}^+ \to \mathbb{R}$ that represents a spreading wave. This wave starts at a low value and reaches a maximum before returning back to a low value. Let $f_c$ be a threshold value. For each point in the domain we define a $T_{\text{start}}$ and $T_{\text{end}}$ by:

$$T_{\text{start}}(x, y) = \{t > 0 | F(x, y, t) = f_c \text{ and } \frac{dF}{dt} > 0\}$$

$$T_{\text{end}}(x, y) = \{t > 0 | F(x, y, t) = f_c \text{ and } \frac{dF}{dt} < 0\}$$

We can then define the unique point that a maximum value is attained in between each of these times:

$$T_{\text{peak}}(x, y) = \{T_{\text{start}}(x, y) < t < T_{\text{end}}(x, y) | F(x, y, t) \text{ is maximum}\}$$

This is a multivalued function, but each branch is part of a continuous function in $(x, y)$. For each point $(x, y)$, we can define the speed of the wave that is passing at that time as:

$$v_{\text{peak}}(x, y) = \frac{1}{|\nabla(T_{\text{peak}}(x, y))|}$$

. For the entire domain, the velocity is the average of the above expression over all space and time.

### S2b: Duration

The duration is calculated in a slightly different fashion in 1D and 2D. In 1D, we take all of the ionic concentrations and scale them so that they vary between 0 and 1. We then take an average of these values. The duration is given by the time over which this value is above 0.1.

For the 2D spiral, we use just the neuronal membrane voltage. We define duration to be the time the neuron is depolarized above $-50$mV.

## S2c: Energy

Here we include a short discussion of the energy balance of the model. For full details, we refer the reader to [1]. Let $G$ be the free energy of the system:

$$G = \int_\Omega \left( \sum_{k=1}^{3} \left( RT \left( a_k \ln\left(\frac{a_k}{\alpha_k}\right) + \sum_{i=1}^{M} \alpha_k c_i^k \ln\left(c_i^k\right) \right) \right) + \sum_{k=1}^{2} \frac{1}{2}\gamma_k C_{\mathrm{m}} \phi_{k\mathrm{e}}^2 \right) dx.$$

The free energy is thus a sum of the entropic contributions from ions and impermeable solutes as well as the electrostatic energy stored in the membrane capacitor. Provided the ionic concentrations $c_i^k$ and $\phi_k$ satisfy the multidomain electrodiffusion model, the free energy satisfies the equation:

$$\begin{aligned}
\frac{dG}{dt} &= -I_{\mathrm{bulk}} - I_{\mathrm{mem}} - I_{\mathrm{bath}}, \\
I_{\mathrm{bulk}} &= \int_\Omega \left( \sum_{k=1}^{3} \sum_{i=1}^{M} \frac{D_i^k c_i^k}{RT} |\nabla \mu_i^k|^2 \right) dx, \\
I_{\mathrm{mem}} &= \int_\Omega \left( \sum_{k=1}^{2} \gamma_k \left( \psi_{k\mathrm{e}} w_k + \sum_{i=1}^{M} \mu_i^{k\mathrm{e}} g_i^k \right) \right) dx, \\
I_{\mathrm{bath}} &= \int_\Omega \sum_{i=1}^{M} \mu_i^{\mathrm{e}} f_i^{\mathrm{bath}} dx.
\end{aligned}$$

The free energy difference can be written as a sum of three terms, which represent the free energy dissipation or input coming from the bulk solvent (intracellular and extracellular spaces), the membrane, and the interaction with the bath. In the above, $\mu_i^{kN}$ is the chemical potential difference with $\mu_i^k = RT\left(\ln(c_i^k + 1) + z_i F c_i^k \phi_k\right)$, and $\psi_k$ is the water potential $\psi_k = -RT\left(a_k/\alpha_k + \sum_{i=1}^{M} c_i^k\right)$. Excluding the possible free energy input from the external bath, the only free energy input into the system is through ionic pumps, which contributes part of the term $I_{\mathrm{mem}}$, which can be expressed as:

$$I_{\mathrm{mem}}^{\mathrm{pumps}} = \sum_{k=\mathrm{n,g}} -\gamma_k \left( 2h_{\mathrm{NaK}}^k \left( RT \log\left(\frac{c_{\mathrm{Na}}^k}{c_{\mathrm{Na}}^{\mathrm{e}}}\right) + F\phi_{k\mathrm{e}} \right) - 3h_{\mathrm{NaK}}^k \left( RT \log\left(\frac{c_{\mathrm{K}}^k}{c_{\mathrm{K}}^{\mathrm{e}}}\right) + F\phi_{k\mathrm{e}} \right) \right).$$

This may be equated with the ATP consumption by the NaK ATPase, up to an efficiency factor relating ATP consumption with the actual free energy that is converted into the concentration differential.

# References

[1] Y. Mori, *A multidomain model for ionic electrodiffusion and osmosis with an application to cortical spreading depression*, Physica D: Nonlinear Phenomena, 308 (2015), pp. 94–108.
