# Supplementary material for: A computational study on the role of glutamate and NMDA receptors on cortical spreading depression using a multidomain electrodiffusion model
Source: PLoS Comput Biol. 2019 Dec 2;15(12):e1007455. doi: 10.1371/journal.pcbi.1007455 (PMC6907880; doi:10.1371/journal.pcbi.1007455)
Supplement: S3 Text — Links to the simulation code are provided. (PDF) [file pcbi.1007455.s003.pdf]

Supporting Information 3 for: A computational study on the role of glutamate and NMDA receptors on cortical spreading depression using a multidomain electrodiffusion model

Austin Tuttle Jorge Riera-Diaz Yoichiro Mori

S3: Code

The code used to perform these simulations is available on GitHub. A more user friendly version of this code that readily generates 2D plane waves is at `https://github.com/ADTuttle/2d_csd_lite` and can be modified to generate spiral waves. The code used to generate spiral waves (and more) is at `https://github.com/ADTuttle/2d_csd` on the `update_spiral_work` branch and requires careful setup. To visualize the output data files in Matlab `https://github.com/ADTuttle/2d_csd_visualizer` provides a simple GUI.
