# Supplementary material for: A computational study on the role of glutamate and NMDA receptors on cortical spreading depression using a multidomain electrodiffusion model
Source: PLoS Comput Biol. 2019 Dec 2;15(12):e1007455. doi: 10.1371/journal.pcbi.1007455 (PMC6907880; doi:10.1371/journal.pcbi.1007455)
Supplement: Attachment — Submitted filename: response.pdf [file pcbi.1007455.s004.pdf]

Response to Reviewer Comments

Austin Tuttle, Jorge Riera Diaz and Yoichiro Mori

September 14, 2019

We thank the two reviewers for their careful reading of our manuscript. We believe that our manuscript is much improved. In the revised manuscript, the revisions are given in red.

Reviewer #1: This paper studied the role of glutamate and NMDA receptor dynamics in an electrodiffusion model of cortical spreading depression (SD) in both 1D and 2D spatial dimensions. They found that SD propagation depends on two distinct but overlapping mechanisms: 1) NaP driven SD propagation relies primarily on extracellular K+ diffusion; 2) NMDAR driven SD propagation depends on glutamate diffusion. For the first time, the inverted saddle signature of the extracellular voltage shift during SD can be explained by the coexistence of these two mechanisms: the first valley corresponds primarily to NaP activation and the latter valley to NMDAR activation. They also studied the properties of the spiral waves in 2D models. They found that the higher the NMDAR expression, the higher the overall energy consumption, which indicates the potential neuroprotective effect of NMDAR antagonists in recurring SD.

We thank the reviewer for the positive assessment of our paper.

Overall, the article is well organized and presented. However, the following revisions should be considered:

1. About the description in Table 1 and 2, the authors should keep the first letter of second word either lowercase or uppercase consistently. The figure legends and subsection titles also have similar issues.

   This point has been fixed. (Q. to Austin)

2. Page 9 from line 162 to 172, for the readers who are not using PETSc software package, its hard to understand these terms. It would be better if the authors explain a little bit more about them.

   We thank the reviewer for pointing this out. Whether using PETSc or not, solving large nonlinear systems typically involves a) the general algorithm used for the nonlinear equation b) linear solver, and c) preconditioners. This general framework is explained in a sentence we added in this section. Some of the routines that we list here are standard numerical linear algebra routines (such as GMRES, incomplete LU) that are discussed in textbooks of numerical linear algebra, for which we have added a citation. We believe

that the details of some of the routines detract from the main thesis of the paper, and thus refer the reader to the thesis of the first author or the PETSc manual.

3. Page 10 from line 201 to 204, too many where, and its not quite easy to understand this sentence.

   Thank you for pointing this out. This has been fixed.

4. What is the advantage of using electrodiffusion model rather than Hodgkin-Huxley-type model?

   This is an important point that we failed to adequately explain. Hodgkin-Huxley type models are suitable for fast electrophysiological activity, during which ionic concentration fluctuations are negligible. SD is a relatively slow phenomenon accompanied by large ion concentration changes. In many SD models, reaction-diffusion type systems are used instead, essentially treating ions as uncharged, but this fails to capture important biophysics (including the extracellular voltage deflection). In order to properly model SD, an electrodiffusive framework is necessary. This is now explained in the Introduction of the paper.

5. About energy consumption, it is not clear how did the author gets the equation (24) and why dG/dt=-Ibulk-Imem. What is the difference of the energy consumption here and the ATP energy consumption due to the pump?

   Section S5 has been rewritten to give a better presentation of the free energy calculation. The free energy equation can be obtained by manipulating the main set of equations, the details of which can be found in previous papers by the last author. The energy consumption here is essentially the same as ATP energy consumption by the pumps, except that the two differ by an unknown efficiency ratio equal to the fraction of the actual ATP hydrolysis energy that was converted to a transmembrane ionic concentration differential.

Reviewer #2 (Geir Halnes): Review of the manuscript: A computational study on the role of glutamate and NMDA receptors on cortical spreading depression using a multidomain electrodiffusion model.

The authors present a three-domain continuum model of neural tissue susceptible to spreading-depression (SD). The model includes a neuronal, glial and extracellular domain, and accounts for electrodiffusive ion transport within the domains, as well as for ion exchange between the domains via a set of ion channels, ion pumps and NMDA synapses. The simulations explore in a convincing way the differences and interplay between the propagation of the SD wave predicted by (1) persistent sodium activation + K+ diffusion, and (2) NMDA activation + Glutamate-diffusion, in a way which can explain various experimental observations. This is done both for a 1D planar wave and for 2D-spiralling waves (the latter of which this model is the first ever to simulate).

A model like this should be welcomed by the neuroscience community as a tool for exploring and testing hypothesis regarding the mechanisms underlying the pathophysiology of SD.

The modelling work seems well conducted, and I would much like to see it published in PLoS CB. There are, however, some improvements that I think should be made to the manuscript beforehand, mainly in terms of how the model is introduced, how the modelling assumptions are discussed, and how the simulations are related to the biological system/phenomenon at hand. I think this would amount to a minor to moderate revision.

We thank Geir for his positive assessment of and his meticulous reading of our manuscript. As a result of his suggestions, we believe the manuscript has much improved.

Moderately Major points:

1. Relating to biology

    (a) I miss in the introduction a brief overview of the neurophysiology of SD. One thing that confuses me a bit is the role of Nap in this and previous models of the authors, and also the previous (cited) model by Kager et al. This may reflect my lack of knowledge, but I thought that the common view of SD in relation to the K+ diffusion hypothesis was that the membrane depolarization came from AP-firing (e.g., via Nat and Kdr) and the following increase in the K+ reversal potential due to a gradual increase in extracellular K+ (see e.g., Ayata & Lauritzen 2015). There are probably good reasons to exclude APs in a coarse-grained model like this. However, should Nap be understood as a playing the role as a stand-in mechanism for Nat, e.g. does Nap sort of work as a temporally averaged version of Nat on a long time scale, or is it in itself the key mechanism? That is, is the depolarization (in biology and in the model) explained mainly by a persistent sodium current or by K+ reversal potential changes which could follow from AP firing?

    This is an interesting point, and we thank the reviewer for raising this issue. The role of fast Na currents in initiation of SD is exactly right. The problem is that fast Na channels inactivate very quickly. It is thus difficult in theoretical models that only have fast Na currents to sustain a prolonged depolarization that will lead to a K flood. To address this issue, Kager et.al. use the persistent Na current, which is known to exist but whose prevalence in the CNS may not be as well-established. An alternative is that NMDA receptors take on the role of maintaining a prolonged depolarization; NMDA receptors are known to have long inactivation kinetics. This point is now discussed in Section 2.1.

    (b) I will not demand it, but I think a schematic figure 1 that illustrates what goes into the model would help the reader to get into the material.

This is now included.

(c) Fig. 1 is introduced with (line 143-144): "Since S is linear in s we can directly solve the above equations. Fig. 1 shows time profiles of an example 1D simulation". I think the simulation should be explained more carefully to help the reader get into the material. How was SD triggered in the system? Was there an input signal? What was the recording position?

The wave is initiated by a transient increase in neuronal membrane permeability. This is now explained at the end of Subsection 3.1. The recording position is also mentioned in the caption of Fig. 1.

(d) Likewise, the spiral simulations (line 242-243) were introduced with: "In two spatial dimensions, we can obtain more interesting dynamics. In the following, we focus on spirals. To create a spiral, we first create an electrophysiologically refractory region in the center of the computational domain." The way this is written makes it appear like the spirals were created "just for fun". I would suggest that this subsection is introduced in a more biology-oriented fashion, i.e. by briefly explaining that such spirals have been observed in biological systems, and as such motivating why one would like to simulate them.

This is now explained at the beginning of Section 4.3. We believe that spirals are important to study because it is self-sustaining, likely associated with pathophysiology, and suggests links to cardiac electrophysiology.

(e) Relating to the above quote, it is unclear to me how "an electrophysiological refractory region" was created in the center of the computational domain, and what it represents biologically. This must be more carefully explained.

We transiently abolish the activating NaP and NMDAR currents, and initiate the wave as in the 1D simulation. This is now explained at the beginning of Section 4.3.

(f) Line 279-281 read: "We also note that the range of parameter values of Nap and NMDAR for which a spiral does not form is much larger than the corresponding range of propagation failure for 1D planar wave. Given the recurring nature of the SD spiral, a higher expression level of the active currents are needed for its generation." This is a nice model prediction. Can it be tied to some experimental observations, or discussed in terms of where/when we see spirals or planar waves?

This is a difficult question for which we do not have a definitive answer. At the end of Section 4.4, we state that this implies that only cortical areas highly susceptible to SD would experience SD spirals.

(g) Generally (like in my points 1.3-1.5), the simulated results could be described in some more detail, and could be related more strongly to the biological scenario that is being simulated. Such improvements

are likely to increase the impact that this work can have on the community.

We appreciate the reviewers comment. We hope that the modifications made above will serve this purpose.

(h) In Fig. 13, for small distances, the Average work is larger than the Maximum work. This seems wrong. Is it something here that I don't understand?

The plot here was confusing; y-axis for the two plots are different, which is now emphasized in the figure caption.

2. Eletrodiffusive continuum model

By necessity, a coarse grained model like this rests on a series of assumptions regarding how the detailed structures and morphologies of neuropil can be collapsed into a tree-domain continuum in a meaningful manner. Whereas I believe that most of the assumptions made in the proposed model are sound and motivated, I feel like several of them could be stated and discussed in some further detail.

(a) As suggested by eq. 5 continuous electrodiffusion occurs internally in all three domains. While it has previously been motivated that transport through the extracellular space and through the gap-junction coupled astrocytic syncytium can be represented as a continuums (Chen & Nicholson 2000), I have not seen a corresponding motivation for such continuous transport in neurons. I think this at least should be commented on when introduced, and perhaps given a brief discussion.

The reviewer is absolutely correct; we have failed to adequately explain the connection between gap junctional coupling and the diffusion coefficient. In the simulations, the diffusion coefficient in the neuronal compartment is set to 0. This is now explained in Section 2, right before Section 2.1.

(b) The tortuosity given in Table 4 suggests that the porous medium approximation was used, but the tortuosity does not appear in eq. 5. Was or wasn't it part of the model?

The dependence of the diffusion coefficient on the tortuosity is now discussed in the Appendix in Table 4.

(c) Eq. 4 suggests that the extracellular space interacted with an omnipresent bath solution. This assumption appears somewhat bold, and should be discussed when introduced. Is it a technicality, or does the magnitude of the bath interaction play an important role for the simulation outcome? Was it tuned? What could it represent? Blood vessels, which are not part of the model, or leakage to deeper layers or into white matter?

Thank you very much for pointing this out. There was an error in our presentation of Eq. 3-6, and this is now corrected. As can be

seen in our new presentation, we are effectively linking the whole computational domain with an external bath that is approximately 1cm away. This is sufficiently far away so that the presence of the bath does not materially affect the results of the simulation.

(d) The code for the model should be made available online. I think it has the potential to be a much used tool for exploring SD by many labs, at least if it is moderately easy to download and run the code.

Thank you for this suggestion. We had initially planned to make the code public as soon as the paper was published, but we have decided that there is really no reason to wait for publication. The code is now available through the weblink indicated at the end of the paper. We would be honored if others can use our code.

3. Language Some parts of the manuscript seems hastily written, and the authors should do a revision before the final version is submitted. Especially the figure texts are often very sparse, sometimes should provide more information.

   We have streamlined the language. We hope that the presentation is now clearer.

Minor Points

- Fig 6: Each graph is 4 different NMDAR should read: Each panel shows four different NMDAR.

  This has been corrected.

- Fig 10: have very wide arcs that the main drivers do not have: I was not able to understand this from looking at the figure. I think the arcs should be explained more carefully in the main text, since these results are not easy to wrap ones head around.

  This statement was misleading and has been eliminated.

- Fig 11: “Period of each point, time between each depolarization for each point in the domain.” This should be made into a sentence and related explicitly to something that we see in the figure.

  This has been corrected.

- Fig. 13: by averaging around the spiral center. Should this be averaging over a circle around the spiral center? Also: with a smaller increasing should be with a smaller increase.

  This has been modified.

- Line 46: in either 1D or 0D. Whereas I understand what 0D means, it seemed a bit odd. Perhaps explain that this is about point models with no spatial extension?

- Eq. 3-5. Is it necessary to use two notations (1,2,3 or n,g,e) for index k? It would be tidier to use n,g,e consistently.

  This has been corrected.

- Line 116-117: Typo: Bg greater than Bg. Line 198: An important difference between the two modes of propagation. I think you should define here what the two modes are.

  This has been modified.

- Line 303: NMDAR driven propagation depends on glutamate diffusion and is strongly influenced by volume changes. Is this not the case for K+ diffusion? Please comment!

  This refers to our study in Section 4.2 on NMDAR mediated propagation. Volume changed do not seem to have the pronounced effect for NaP/K diffusion although we have not explored this in depth.

- Line 337: Fast Na+ currents will require very fine time-stepping, which will lead to further challenges in the numerical method. Again, I wonder here if Nap could essentially have the same long term effect as a (temporally smeared) seizure. If appropriate, this could be discussed here. Also, I suppose that the inclusion of fast Na-currents in a continuum-model is also poses some conceptual problems? For example, in 1D, does it not effectively correspond to the assumption that all neurons (at a given x) fire completely synchronous APs?

  The comment on NaP was addressed earlier. The reviewer's comment on synchrony is very interesting and significant. This is now included in the Conclusion section.
